# SARS-CoV-2 in the Middle Ear-CovEar: A Prospective Pilot Study

**DOI:** 10.3390/jpm13060905

**Published:** 2023-05-28

**Authors:** Nina Rubicz, Nikolaus Poier-Fabian, Christian Paar, Markus Winkler-Zamani, Philipp Hermann, Stefan Raidl, Paul Martin Zwittag

**Affiliations:** 1 Department of Otorhinolaryngology, Head and Neck Surgery, Kepler University Hospital GmbH, Krankenhausstrasse 9, 4020 Linz, Austria; nina.rubicz@kepleruniklinikum.at (N.R.); nikolaus.poier-fabian@uniklinikum.at (N.P.-F.); 2 Medical Faculty, Johannes Kepler University Linz, Altenbergerstrasse 69, 4040 Linz, Austria; 3 Institute of Laboratory Medicine, Kepler University Hospital GmbH, Krankenhausstrasse 9, 4020 Linz, Austria; christian.paar@kepleruniklinikum.at; 4 Institute for Pathology and Microbiology, Kepler University Hospital GmbH, Krankenhausstrasse 9, 4020 Linz, Austria; markus.winkler-zamani@kepleruniklinikum.at; 5 Center for Clinical Studies (CCS Linz), Johannes Kepler University Linz, Krankenhausstrasse 5, 4040 Linz, Austria; philipp.hermann@jku.at (P.H.); stefan.raidl@jku.at (S.R.)

**Keywords:** COVID-19, coronavirus, SARS-CoV-2, middle ear, ear surgery, middle ear surgery, otologic surgery

## Abstract

In post-mortem analyses, SARS-CoV-2 was found in the middle ear of some, but not all, patients with COVID-19. It is not clear whether SARS-CoV-2 penetrated the ear passively post mortem, or existed in the middle ear of living patients during, and perhaps also after, infection. This study investigated whether SARS-CoV-2 can be found in the middle ear of living patients during ear surgery. Swabs from the nasopharynx, the filter connected to the tracheal tube and secretions from the middle ear were collected during middle ear surgery. All samples were tested for the presence of SARS-CoV-2 using PCR. History of vaccination, COVID-19 history and contact with SARS-CoV-2-positive individuals were recorded preoperatively. Postoperative SARS-CoV-2 infection was noted at the follow-up visit. Overall, 63 participants (62%) were children and 39 (38%) were adults. SARS-CoV-2 was found in the middle ear and in the nasopharynx of two and four CovEar study participants, respectively. The filter connected to the tracheal tube was sterile in all cases. Cycle threshold (ct) values of the PCR test were between 25.94 and 37.06. SARS-CoV-2 penetrated the middle ear of living patients and was found in asymptomatic patients. The presence of SARS-CoV-2 in the middle ear may have implications for ear surgery and can pose a risk of infection for operating room staff. It may also directly affect the audio–vestibular system.

## 1. Introduction

Almost 615 million cases of coronavirus disease 2019 (COVID-19) have been recorded at the time of writing this manuscript. In about 6.5 million people, COVID-19 led to death [1]. COVID-19 is caused by the severe acute respiratory syndrome coronavirus 2 (SARS-CoV-2) and has been linked to acute otitis media (OM) [2], as well as longer term otologic sequelae including hearing loss and tinnitus [3]. From the beginning of the pandemic, it was known that the virus named SARS-CoV-2, which is responsible for this disease, spreads by droplet transmission and has a reservoir in the upper aerodigestive tract [4]. The nasopharynx is almost universally targeted for swab collection in the diagnosis of COVID-19 because of the high viral load there. Swabs from the nasopharynx followed by detection of the viral genome using reverse transcription polymerase chain reaction (PCR) are therefore considered to be the gold standard for SARS-CoV-2 detection [5]. Although the nasal cavity is a known viral entry site, there is little data as to whether the mucosal epithelium lining the middle ear is infected and if so, if there are resulting clinical implications. Due to the anatomical connection between the nasopharynx and the middle ear through the Eustachian tube, it is assumed that the middle ear and the mastoid air cell system may become contaminated with SARS-CoV-2 in COVID-19 [6]. Therefore, the middle ear and the mastoid are considered to be potentially infectious spaces. In particular, ear surgery of long duration, or the spreading of droplets and aerosols when drilling or suctioning seems to pose a potential risk for the operating room personnel. At the beginning of the pandemic, a hypothesis was advanced that the middle ear and mastoid air cell system might be colonized with SARS-CoV-2 in positive patients, and it was recommended to delay non-urgent otologic surgery in these patients [6,7]. Several researchers reported cases of otitis media in a COVID-19 patient or the isolation of SARS-CoV-2 from mastoid and middle ear specimens in deceased COVID-19 patients [2,8]. Therefore, ENT doctors have been warned about ear surgery in patients with unknown SARS-CoV-2 status [6].

To date, SARS-CoV-2 was found post mortem in the middle ear of some, but not all, patients with COVID-19 [8,9,10,11]. It is not clear whether SARS-CoV-2 penetrated the ear passively post mortem or existed in the middle ear before death.

The aim of our study was to investigate the presence of SARS-CoV-2 in the middle ear of living patients. If SARS-CoV-2 is found in the middle ear of living patients, even more serious questions arise: 1. Is it present in patients with a severe course of disease only, or is it in asymptomatic patients too? 2. Can SARS-CoV-2 be found in the middle ear of vaccinated patients? 3. Does the middle ear constitute a reservoir for SARS-CoV-2? 4. Is SARS-CoV-2 in the middle ear a threat to the ear surgeon and operating room staff?

## 2. Materials and Methods

### 2.1. Study Design, Inclusion Criteria and Ethics Approval

The present monocentric, prospective study was performed between March 2021 and March 2022. Patients older than 1 year who underwent middle ear surgery in our department of otorhinolaryngology (ORL) and agreed to participate in the study were included. We took into account both elective and acute ear surgeries with opening of the middle ear. Paracentesis (PC), tympanic drainage (TD), tympanoplasty with and without mastoidectomy, stapesplasty, cochlear and vibrant soundbridge implantation were taken into account. All indications and operations were conducted by three experienced ORL doctors. The exclusion criteria were age less than 1 year and no informed consent to participate in the study. The study protocol was approved by the Johannes Kepler University Ethics Committee in accordance with the Declaration of Helsinki (EK Nr: 1026/2021). Written consent was obtained from all participants.

### 2.2. Material

#### Clinical Data

Age, weight, sex, indication and type of surgery, previous illnesses, history of vaccination and any contact with people who tested positive for SARS-CoV-2 were recorded preoperatively. At the follow up visit 3 months after surgery, patients were additionally asked about SARS-CoV-2 infections in the postoperative period. We also recorded the number of all middle ear operations in Department of Otorhinolaryngology, Head and Neck Surgery, Kepler University Hospital, separately for children and adults and the number of SARS-CoV-2-positive patients in our country and our region during the study period [12]. All study participants were tested prior to hospitalization and had to be negative for surgery.

### 2.3. Sample Collection

In each patient, a swab from the nasopharynx as well as secretions from the middle ear were collected during surgery. After the surgery, another swab was taken from the filter between the tracheal tube and respirator.

In children who underwent ear surgery with concomitant pharyngeal surgery such as tonsillectomies, tonsillotomies or adenotomies, samples from the nasopharynx were collected through the mouth under direct vision using a mirror to avoid contamination. In adults and children who underwent isolated ear surgery, swabs from the nasopharynx were taken through the nose during surgery. The virus stabilization Vacuette Tube, 3 mL (Greiner Bio-One, Kremsmünster, Austria), was used, with specimen collected using nasopharyngeal flocked swabs (iClean^®^, Chenyang Global, Yangzhou, China).

Middle ear secretions were collected immediately after opening the middle ear. If there was no accumulation of fluid, the middle ear was irrigated using 1 mL of sterile normal saline and the resulting specimen was collected. Afterwards, 1 mL of saline was used to rinse the area. In bilateral operations, material was obtained from both ears, mixed in a sample container and analyzed together. A sterile tracheal suction trap (Primed^®^) combined with a conventional suction system was used to collect ear secretions. The biological materials were sent to the laboratory immediately after the operation.

### 2.4. Laboratory Procedures

For molecular analysis, aliquots were frozen at minus 80 °C to be batch processed later for nucleic acid extraction and analysis. The QIASymphony Virus/Pathogen Midi Kit (Co., Qiagen, Hilden, Germany) was used for nucleic acid extraction, whereas for detection the Real-Time PCR SARS-CoV-2 Winterplex assay (genesig^®^, Primerdesign, UK) was utilized in conjunction with the Cobas z480 Real-Time Analyzer.

### 2.5. Statistical Analysis

Descriptive statistics are provided for all observations with the groups of children and adults shown separately. For nominal variables, absolute and relative frequencies were computed. Non-normal metric and ordinal variables are described with median and interquartile range (median (IQR)). The normality of metric variables was tested using the Shapiro–Wilk test (SW). Associations between nominal variables or ordinal variables with few observations were analyzed using Fisher’s exact test based on 100,000 Monte Carlo simulations. For metric and ordinal variables, the Mann–Whitney U test was used to test differences across the study groups. The level of significance was set at 0.05 and tests were conducted as two-sided. Statistical analyses were conducted using the statistical software package R [13].

## 3. Results

### 3.1. Demographics

**Figure 1:**
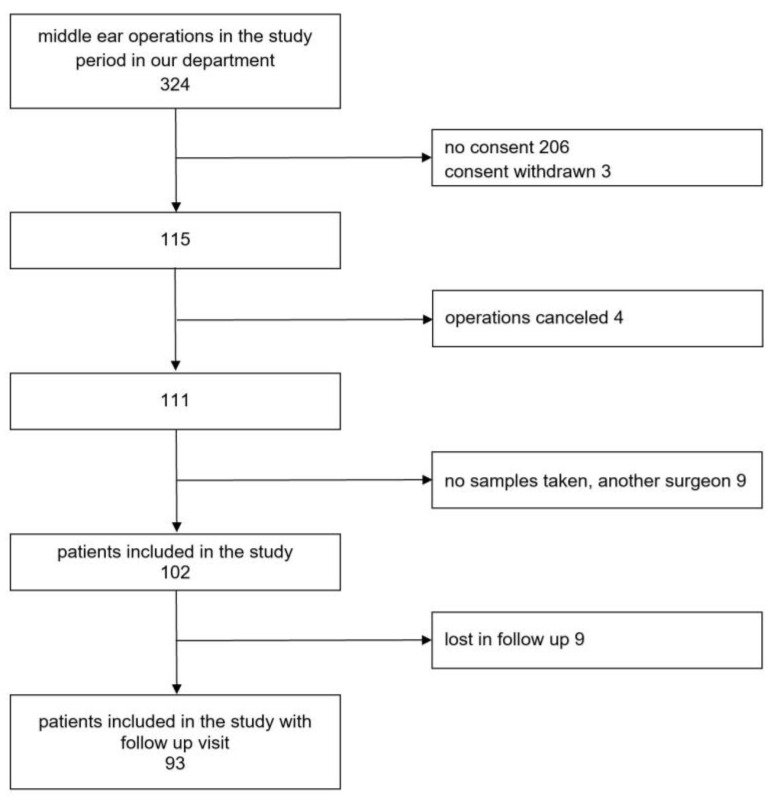
Flowchart of participant recruitment.

**Figure 2:**
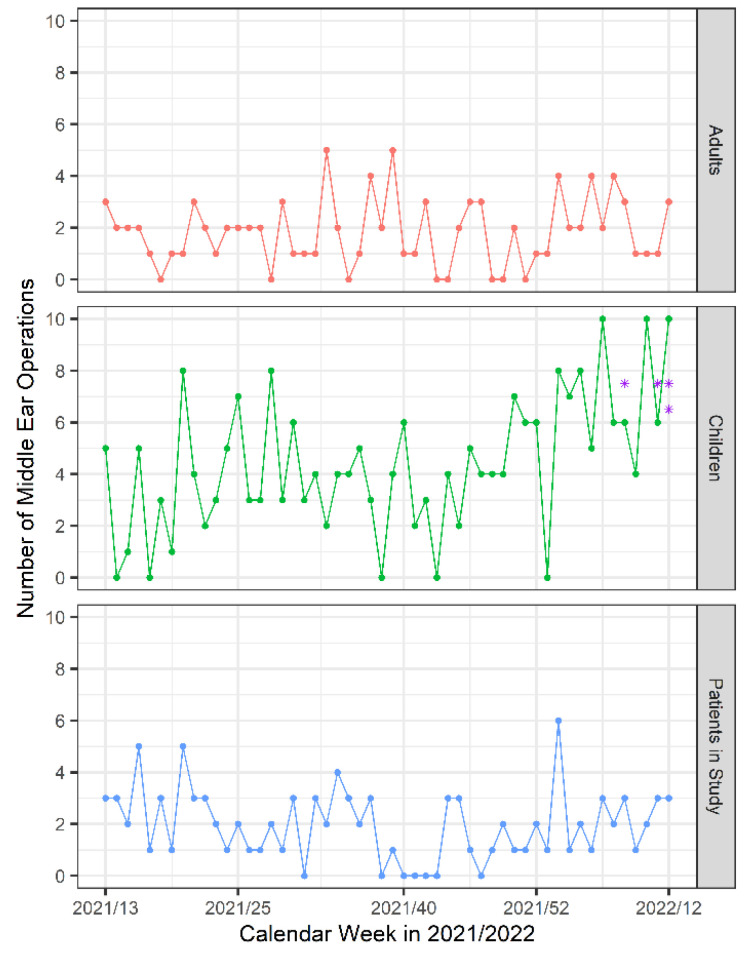
Number of middle ear operations in children and adults and number of patients enrolled in the study per calendar week in 2021/2022. (*—patients with positive SARS-CoV-2 PCR tests in the middle ear and/or nasopharynx at the time of surgery).

From the 324 patients who underwent middle ear surgery in our department, 115 signed the information sheet and informed consent and were enrolled in our study. Operations were canceled for four patients. Another nine patients were excluded from the study because the surgery was performed by a surgeon who was not part of the research team, or due to failure in sample collection (Figure 1). The number of all middle ear operations in our ENT department in children and adults during the study period and number of patients enrolled in the study are shown in Figure 2.

The group characteristics of the 102 patients who were ultimately included in the study are listed in Table 1. A follow-up visit was reported three months postoperatively for 91.18% of the study participants. The median age at surgery was 5.5 years (range, 1–94 years, IQR of 43 years), 63 participants (61.76%) were children and 57 (55.88%) were male.

To better understand whether there are factors that affect colonization of the mastoid and middle ear in SARS-CoV-2-related chronic diseases, information on disorders and the American Society of Anaesthesiologists (ASA)’s classification of Physical Health are shown in Table 2. The ASA score was grade I in 70 patients (68.63%) and grade II in 29 patients (28.43%). Scores IV to VI were not recorded at all. Statistically significant differences between the group of children and adults were found regarding the occurrence of cardiovascular disease, respiratory disease, gastroesophageal reflux, and regarding the ASA-Score.

### 3.2. SARS-CoV-2

The epidemiological data is presented in Table 3. In our group, 25 patients (24.51% of all patients) were vaccinated against SARS-CoV-2, including two children (1.96%). Thirteen (12.75%) patients had had COVID-19 and 34 (33.33%) had proven contact with a SARS-CoV-2-positive person at any time prior to enrolment in the study. The number of unvaccinated patients in these groups was 11 (10.78%) and 28 (27.45%), respectively.

From 102 study participants, 2 (1.96%) had positive SARS-CoV-2 PCR tests in both the middle ear and the nasopharynx. Two (1.96%) further participants had positive SARS-CoV-2 PCR tests in the nasopharynx only, but not in the middle ear.

Ten of the intraoperatively negative patients had had COVID-19 preoperatively: a minimum of 8 days and a maximum of 495 days were counted from infection to surgery, with a median time of 166.5 days. Proven contact with SARS-CoV-2-positive persons was reported in 32 patients before operation: the minimum was 9 days, the maximum 667 was days and the median time was 129 days.

All SARS-CoV-2-positive patients were children who had an elective operation. The characteristics of the SARS-CoV-2-positive patients are presented in Table 4. They had no chronic diseases or disorders. The health status of all of these patients was assessed as ASA score I. None of the four children were vaccinated against SARS-CoV-2. Three of them had COVID-19 preoperatively occurring 7, 50 or 51 days before surgery. Moreover, two of them had contact with SARS-CoV-2-positive people preoperatively, 15 or 68 days before surgery. None of the four children had COVID-19 symptoms before, during or after the operation. Cycle threshold (ct) values of the PCR test were between 25.94 and 37.06.

**Figure 3:**
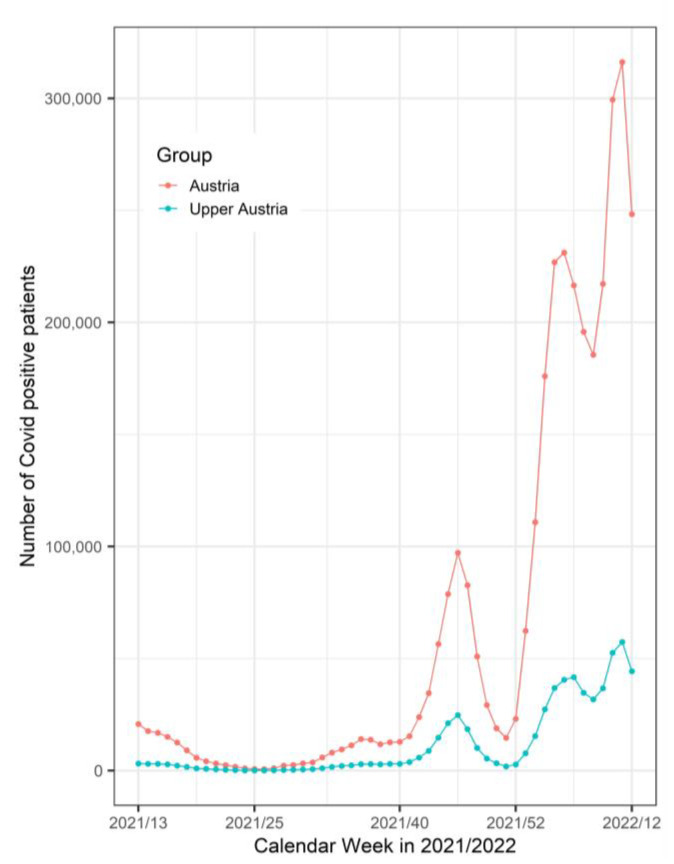
Number of COVID-19-Positive Patients in Austria and Upper Austria per Calendar Week in 2021/2022.

Figure 3 shows the number of SARS-CoV2-positive patients in Austria and in our region of Upper Austria during the study period.

## 4. Discussion

The connection between the upper respiratory tract and the middle ear via the Eustachian tube poses the possibility for viruses to enter the middle ear spaces. Three post-mortem studies confirmed the presence of SARS-CoV-2 in biopsies from the middle ear [8,9,10]. Furthermore, physiological research indicates that the mucosal lining of the middle ear can be infected by SARS-CoV-2. Both middle ear and nasal epithelial cells show relatively high expression of angiotensin-converting enzyme 2 (ACE2), required for SARS-CoV-2 entry [9,14]. In particular cases, attempts were made to examine the middle ear and mastoid in living patients, but no SARS-CoV-2 could be detected [15]. It was not clear whether SARS-CoV-2 penetrated the ear passively post mortem or existed in the middle ear before death. Three post-mortem studies were conducted in corpses who were SARS-CoV-2 positive and met COVID-19 criteria before death. Middle ear samples were positive in 67% (n = 2/3) and 50% (n = 3/6 and n = 4/8) of these cases [8,9,10]. Additionally, two of these studies performed nasal swabs, which were positive in 50% and 75% of cases, respectively [9,10]. All cases with positive results from the ear also had positive results from the nasal swabs. Frazier et al. investigated SARS-CoV-2 presence in the middle ears and mastoids separately in three corpses [8]. In one corpse, SARS-CoV-2 was found only in one middle ear, whereas the other middle ear and both of the mastoids were virus free. The second corpse was positive for SARS-CoV-2 in both middle ears and both mastoids. The third corpse had no virus in either the middle ears or mastoids. The post-mortem interval was 44 h for the COVID-19-negative case, 48 h for the COVID-19-positive case in one middle ear and 16 h for the COVID-19-positive case in both middle ears and mastoids. The post-mortem times were suspected as an important factor for the various findings [8]. In another study, the problem of post-mortem time differences was resolved. In each examined case, the post-mortem time was limited up to 3 h. The same SARS-CoV-2 test result, either positive or negative, from the ear and nasopharynx was obtained from all decedents in this study. [9]. Differences in SARS-CoV-2 presence in the middle ear between patients dying from COVID-19 and dying with COVID-19 was suspected but there is no answer for this question thus far [8]. To the best of our knowledge, the CovEar study is the first work to investigate the presence of SARS-CoV-2 in the middle ear, using samples taken during ear surgery of living patients. None of the CovEar study participants had infection symptoms before, during or after the operation. Although the study group included people without an upper age limit, the virus was detected only in children aged 4 to 8 years in the middle ear or the nasopharynx. The studies conducted thus far were performed in adults only [8,9,10].

All the children who tested positive in the CovEar study, either in the middle ear or the nasopharynx, had an asymptomatic course of disease at the time of operation. We analyzed the SARS-CoV-2-positive cases separately. One child with SARS-CoV-2 in the middle ear had proven contact with an infected person 15 days before the operation. The other one had a positive test result 7 days before the operation and a negative result directly before the surgery. The parents of this child did not inform the doctor about the recent infection until the follow-up visit.

The mastoid air cells directly communicate with the middle ear through the aditus and antrum, resulting in a mastoid viral load similar to that of the middle ear mucosa [16]. However, the results published by Frazier et al. [8] showed a lower viral load in the mastoid than in the middle ear. In post-mortem studies, the viral load detected in the middle ear was significantly lower than in the nasal cavity in two of three cases. In the third case, it was higher but not significant [9]. Another post-mortem study confirmed that the virus concentration was highest in the nasopharynx and lowest in the mastoid in three of four cases [8,10]. In our study, the viral load at the time of operation was low in all cases. There was also no consistency when comparing the ct value in the middle ear and the nasopharynx. In one case the ct value in the middle ear was higher (29.77) than in the nasopharynx (25.94), and in the other case the ct value in the nasopharynx (32.15) was higher than in the middle ear (30.03). Our question was whether it is possible for SARS-CoV-2 to exist as a reservoir in the middle ear of SARS-CoV-2-negative individuals. In our group, persons who were SARS-CoV-2 positive in the middle ear were also positive in the nasopharynx.

It is not clear why some people who had COVID-19 preoperatively or had proven contact with SARS-CoV-2-positive people were positive during the operation whereas others were not. In some intraoperative negative cases, the time between proven contact with SARS-CoV-2-positive persons or having COVID-19 and the operation was similar, or even lower than in cases of intraoperative SARS-CoV-2-positive individuals [8]. The study by Frazier et al. confirmed the presence of SARS-CoV-2 in the middle ear and mastoid in post-mortem cases but the study possessed some limitations such as the methodology including the post-mortem interval prior to autopsy. The partial positive results of case 1 and negative results of case 2 may be related to the much longer post-mortem intervals as increased intervals decrease tissue stability and affect viral stability and isolation at autopsy, and rapid autopsy protocols may provide tissues that are more comparable to fresh surgical biopsies. Additional conclusions are limited, particularly regarding asymptomatic carriers and vaccinated individuals and it was proposed that comorbidities may affect middle ear colonization [8]. In the data from the CovEar study, we could not find any indications that additional diseases influence the colonization of the middle ear. Indeed none of the intraoperatively positive individuals were vaccinated, but the number of individuals was too low to propose that the vaccination might influence the result. All positive SARS-CoV-2 cases were diagnosed during the period of time when the number of SARS-CoV-2-positive patients in Austria and in our region of Upper Austria was very high (Figure 3).

In the beginning of the pandemic, many preventive measures and regulations, especially during ear surgery, were introduced to avoid virus transmission. The presence of SARS-CoV-2 in the middle ear was presumed. The drilling and suctioning usually performed during ear surgery can cause droplets and aerosols that spread in the operating room [6,7,17,18]. Furthermore, van Doremalen and coworkers showed that viral particles can remain in the air for at least 3 h [19]. Jewett et al. stated that there is a high risk of generating contaminated aerosols when using high speed drills, which are commonly used in ear surgery nowadays [20]. Consequently, there was a warning about performing ear surgery in patients with unknown status or with a history of COVID-19. The evidence from our study points to the fact that there may be patients in the operating theatre with the virus in the middle ear. Moreover, the confirmation of the presence of SARS-CoV-2 in the middle ear requires more attention to be paid to precautionary measures before procedures on the middle ear are carried out. Due to the direct proximity of the structures of the middle and inner ear, confirmation of the presence of the virus in the middle ear also gives rise to concerns about the penetration of the virus into the structures of the inner ear. Kurabi et al. proposed that SARS-CoV-2, if present in the middle ear, could lead to direct inner ear SARS-CoV-2 infection [9]. During the pandemic, reports about the possible effects of SARS-CoV-2 on various systems of the human body began to appear [21]. El-Anwar et al. reported in their systematic literature review that 2.1% of the subjects suffered from runny nose or rhinorrhea, nasal congestion was detected in 4.1%, effects on smell was documented in 6%, nasal obstruction manifested in 3.4%, sore throat in 11.3%, pharyngeal erythema presented in 5.3%, tonsil enlargement in 1.3%, headache in 10.7%, and upper respiratory tract infection in 1.9% of the patients under investigation [21]. However, the reports very rarely indicated the effects of the virus on the audio–vestibular system. The systematic review by Almufarrij et al. reported multiple cases of hearing loss (e.g., sudden sensorineural), tinnitus and rotatory vertigo in adults with a wide range of COVID-19 symptom severity. Their pooled estimate of prevalence based primarily on retrospective recall of symptoms, was 7.6% for hearing loss, 14.8% for tinnitus and 7.2% for rotatory vertigo. However, it is suspected that these symptoms were not explicitly reported because attention was primarily focused on life-threatening symptoms [3]. After one year into the pandemic, the number of studies reporting audio–vestibular symptoms after COVID-19 have increased [3,22,23,24,25]. It was estimated that the prevalence of vertigo, hearing loss and tinnitus after SARS-CoV-2 infection was 7.2, 7.6 and 14.8%, respectively [25]. However, these symptoms may not only be induced by COVID-19, but also potentially by antiviral medication [3,24]. Moreover, the viral loads detected in symptomatic and asymptomatic patients were similar [26]. Nonetheless, it still remains unclear whether asymptomatic SARS-CoV-2 infections can provoke audio–vestibular symptoms that cannot be subsequently linked with COVID-19. Further investigation is needed in this area.

The main limitation of our study is the relatively small number of cases and their heterogeneity. A further limitation is the small volume of liquid collected in the middle ear. Due to the small amount of test material, it was not possible to repeat the measures. We tried to increase the volume by using fluid from both ears in patients who were operated on both sides.

## 5. Conclusions

The findings of this study show that SARS-CoV-2 penetrates the middle ear of living patients, including asymptomatic individuals. The presence of SARS-CoV-2 in the middle ear can have implications for ear surgery and can pose a risk of infection for operating room staff. It may also directly affect the audio–vestibular system.

## Figures and Tables

**Table 1:** Group Characteristics.

Parameters	Total	Children	Adults
Number of Patients (%)	102 (100)	63 (61.76)	39 (38.24)
Female (%)	45 (44.12)	32 (50.79)	13 (33.33)
Male (%)	57 (55.88)	31 (49.21)	26 (66.67)
Age Median; (IQR) [y]	5.50 (43)	4 (2)	51 (18.50)
Weight Median (IQR) [kg]	25.50 (56.12)	18 (6)	80 (22)
Type of Surgery			
Acute Surgery (%)	3 (2.94)	0 (0)	3 (7.69)
Elective Surgery (%)	99 (97.06)	63 (100)	36 (92.31)
Tympanic Membrane Incision, Tympanostomy Tube Insertion or Removal (%)	75 (73.53)	60 (95.24)	15 (38.46)
Tympanoplasty ± Mastoidectomy (%)	18 (17.65)	3 (4.76)	15 (38.46)
Stapesplasty (%)	3 (2.94)	0 (0)	3 (7.69)
Implantation CI, VSB; (%)	6 (5.88)	0 (0)	6 (15.38)
Indication for Operation			
Otosclerosis (%)	3 (2.94)	0 (0)	3 (7.69)
Hearing Loss (%)	7 (6.86)	0 (0)	7 (17.95)
OME, AOM, Eustachian Tube Dysfunction (%)	67 (65.69)	53 (84.13)	14 (35.90)
CSOM (%)	17 (16.67)	8 (12.70)	9 (23.08)
CHOL (%)	8 (7.84)	2 (3.17)	6 (15.38)

OME = otitis media with effusion, AOM = acute otitis media, CSOM = chronic suppurative otitis media, CHOL = cholesteatoma, CI = cochlear implant, VSB = Vibrant Soundbridge.

**Table 2:** Group Characteristics: General Health Condition.

Parameters	Total (%)	Children (%)	Adults (%)	*p*-Value
ASA Score				
I	70 (68.63)	57 (90.48)	13 (33.33)	
II	29 (28.43)	6 (9.52)	23 (58.97)	<0.001
III	3 (2.94)	0	3 (7.69)	
IV–VI	0	0	0	
Cardiovascular Diseases	15 (14.71)	2 (3.17)	13 (33.33)	<0.001
Reflux	11(10.78)	1 (1.59)	10 (25.64)	<0.001
Respiratory Diseases	8 (7.84)	1 (1.59)	7 (17.95)	<0.005
Obstructive Sleep Apnea	4 (3.92)	3 (4.76)	1 (2.56)	NA
Craniofacial Anomalies	4 (3.92)	2 (3.17) ^a^	2 (5.13) ^b^	NA
Thyroid Gland Disease	4 (3.92)	0 (0)	4 (10.26)	NA
Diabetes Mellitus	3 (2.94)	0 (0)	3 (7.69)	NA
Wegener Disease	1 (0.98)	0 (0)	1(2.56)	NA
Down Syndrome	1 (0.98)	0 (0)	1(2.56)	NA
Muscle Hypotonia	1 (0.98)	1(1.59)	0 (0)	NA
Allergy	9 (8.82)	6 (9.52)	3 (7.69)	1

^a^ Cleft palate and cleft lip and palate. ^b^ Cleft lip and palate, dysmorphia by Down syndrome.

**Table 3:** Epidemiology.

Parameters	Total (%)	Children (%)	Adults (%)
Vaccinated Patients	25 (24.51)	2 (3.17)	23 (58.97)
1. Comirnaty (BioNTech/Pfizer)	19 (18.63)	19 (18.63)	19 (18.63)
2. Jannsen (Johnson & Johnson)	1 (0.98)	1 (0.98)	1 (0.98)
3. Spikevax (Moderna)	1 (0.98)	1 (0.98)	1 (0.98)
1. + 2. Comirnaty + Jannsen	1 (0.98)	1 (0.98)	1 (0.98)
Vaccination of unknown kind	3 (2.94)	3 (2.94)	3 (2.94)
No vaccination	77 (75.49)	77 (75.49)	77 (75.49)
Time between last vaccination and operation			
Min (d)	6	95	6
Max (d)	154	130	154
Median (d)	48		44
IQR (d)	61		59
Mittelwert (d)	62	113	57
NA	1	0	1
Patients with COVID-19 Infection History	13 (12.75)	9 (14.29)	4 (10.26)
Time between the last COVID-19 infection and operation			
Min (d)	7	7	8
Max (d)	495	495	408
Median (d)	132	103	272
IQR (d)	318	140	295
Mittelwert (d)	189	166	240
NA	0	0	0
Patients with Proven Contact	34 (33.33)	27 (42.86)	7 (17.95)
Time between the last proven contact and operation			
Min (d)	9	45	9
Max (d)	667	667	353
Median (d)	129	131	127
IQR (d)	127	129	97
Mittelwert (d)	173	185	130
NA	0	0	0
SARS-CoV-2 in the Ear	2 (1.96)	2 (3.17)	0 (0)
SARS-CoV-2 in the Nasopharynx	4 (3.92)	4 (6.35)	0 (0)
SARS-CoV-2 in the Filter	0 (0)	0 (0)	0 (0)

**Table 4:** Characteristics of SARS CoV-2 Positive Patients.

Parameters	Patient No. 72	Patient No. 95	Patient No. 99	Patient No. 101
Sex and Age [y]	M 4	M 5	F 8	F 4
Weight [kg]	18	20	30	17
Type of Surgery	Elective	Elective	Elective	Elective
Type of Procedure	PC	PC	G	G
Indication for Operation	OME	OME	OME	OME
SARS-CoV-2 in Ear (ct)	+(29.77)	+(30.03)	No	No
SARS-CoV-2 in Nasopharynx (ct)	+(25.94)	+(32.15)	+(32.11)	+(37.06)
SARS-CoV-2 in Filter Sample	No	No	No	No
Vaccination Against SARS-CoV-2	No	No	No	No
COVID-19 Infection Preoperatively [Days Before Operation]	7	No	51	50
Proven Contact with SARS-CoV-2-Positive Person [Days Before Operation]	No	15	68	No
COVID-19 Infection After Surgery	No	No	No	No
Additional Diseases:	No	No	No	No
ASA Score	I	I	I	I

F = female, M = male, PC = paracentesis, G = grommet, OME = otitis media with effusion, +() = positive (ct-value).

## Data Availability

The authors declare that the data from this research are available from the corresponding authors upon reasonable request.
